# The Burden of Human African Trypanosomiasis

**DOI:** 10.1371/journal.pntd.0000333

**Published:** 2008-12-23

**Authors:** Eric M. Fèvre, Beatrix v. Wissmann, Susan C. Welburn, Pascal Lutumba

**Affiliations:** 1 Centre for Infectious Diseases, University of Edinburgh, Ashworth Laboratories, Edinburgh, United Kingdom; 2 Centre for Infectious Diseases, College of Medicine and Veterinary Medicine, University of Edinburgh, Summerhall, Edinburgh, United Kingdom; 3 Programme National de Lutte contre la Trypanosomiase Humaine Africaine, Kinshasa, Democratic Republic of Congo; 4 Institute of Tropical Medicine, Antwerp, Belgium; London School of Hygiene & Tropical Medicine, United Kingdom

## Abstract

Human African trypanosomiasis (HAT, or sleeping sickness) is a protozoan parasitic infection caused by *Trypanosoma brucei rhodesiense* or *Trypanosoma brucei gambiense*. These are neglected tropical diseases, and *T.b. rhodesiense* HAT is a zoonosis. We review current knowledge on the burden of HAT in sub-Saharan Africa, with an emphasis on the disability-adjusted life year (DALY), data sources, and methodological issues relating to the use of this metric for assessing the burden of this disease. We highlight areas where data are lacking to properly quantify the impact of these diseases, mainly relating to quantifying under-reporting and disability associated with infection, and challenge the HAT research community to tackle the neglect in data gathering to enable better evidence-based assessments of burden using DALYs or other appropriate measures.

## Introduction

Human African trypanosomiasis (HAT)—also known as sleeping sickness—is caused by infection with one of two parasites: *Trypanosoma brucei rhodesiense* or *Trypanosoma brucei gambiense*. These organisms are extra-cellular protozoan parasites that are transmitted by insect vectors in the genus *Glossina* (tsetse flies). As with a few other human pathogens (e.g., tuberculosis caused by *Myobacterium tuberculosis* and *M. bovis*), HAT shares the confusion that two different causative organisms cause a similar clinical disease. The parasites can be distinguished through molecular methods [1],[2], but not parasitologically; the geographic range of the parasites has been a key component of the differential diagnosis of HAT, as *T.b. gambiense* occurs in West and Central Africa, and *T.b. rhodesiense* occurs only in East Africa, though there are concerns that an overlap may now have occurred in their ranges [3]. To understand the epidemiology of HAT, as well as its disease and economic burden, it is essential to understand the distinction between the diseases caused by the two parasites.

HAT is restricted to sub-Saharan Africa, in the range of the tsetse vector. The distributions of tsetse (possibly more than 30 species [4] with affinities for specific habitats, although not all species have been confirmed as parasite vectors), and of the parasites within the vector range, are both focal. Thus, HAT is a public health problem where the vector, the parasite (and its reservoir hosts), and humans co-exist.

Here, we review what we know—and, importantly, what we don't know—about the burden of HAT in sub-Saharan Africa, with an emphasis on data sources and methodological issues relating to the use of the disability-adjusted life year (DALY) as a metric for assessing the burden of this disease.

## What We Know about HAT Epidemiology and Burden

*T.b. rhodesiense* is a zoonosis [5],[6], with a number of wildlife [7] and domestic animal species known to act as reservoirs. Where wildlife is not abundant, domestic species, particularly cattle, are the main reservoir [8], with livestock demography driving outbreaks [9]. *T.b. gambiense* is generally not considered zoonotic—it can be isolated from animal hosts [10],[11], but large-scale control campaigns targeting only the human reservoir (active screening and treatment of human cases) are able to locally eliminate transmission [12],[13], and theoretical assessments of control options [14] confirm that from an epidemiological perspective, the presence of animal hosts is unlikely to mean they serve as a reservoir of infection for humans [15] (such hosts and their potential as a source for re-introduction of the parasite to the human population would need to be considered if ever aiming for total elimination of the disease, however). The transmission of HAT occurs primarily in rural areas (with a few exceptions, including peri-urban Kinshasa [16]), in areas at the furthest extremities of the formal health system, creating particular problems for patients to access health care [17],[18], for control campaigns to have an effective outreach [19], and, importantly, in the assessment of the burden of infections, hindering efforts to collect data on how many people are at risk, how many people are infected, and what the impact of the disease is on the social environment. These are not issues restricted to HAT (of either form), but are general among many of the neglected tropical diseases and neglected zoonotic diseases [20],[21].

**Figure 1:**
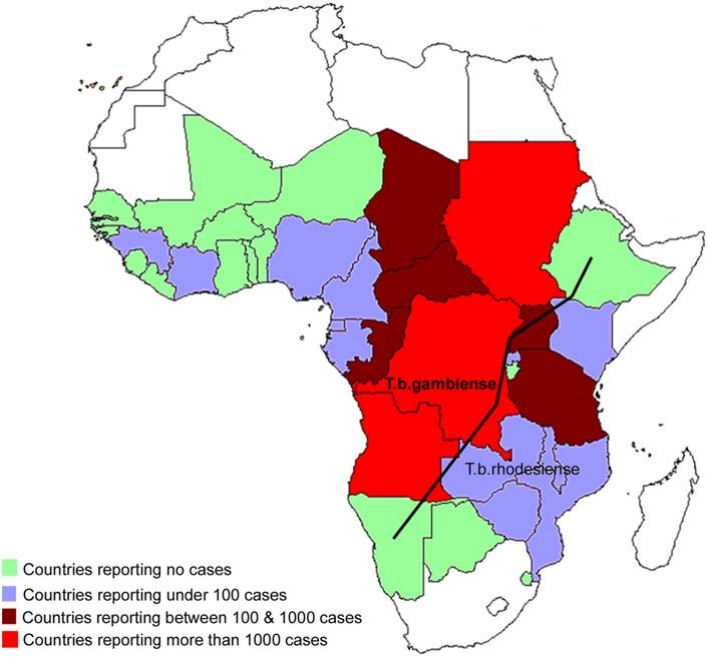
Map of Sleeping Sickness. Image credit: [23].

The available estimates for HAT indicate that 60 million people are at risk (both forms combined) in sub-Saharan Africa [22] (though the evidence base for this figure is questionable and is currently being revised; see http://www.who.int/trypanosomiasis_african/country/en/) in approximately 250 distinct foci (see Figure 1) (a focus is loosely defined as “a zone of transmission to which a geographical name is given” [22]). The greatest burden of *reported* cases is due to *T.b. gambiense*, with 23,832, 19,901, 17,036, 15,651, and 11,382 *T.b. gambiense* and 655, 514, 580, 727, and 486 *T.b. rhodesiense* cases in 2002, 2003, 2004, 2005, and 2006, respectively [23],[24]. Approximately two-thirds of reported *T.b. gambiense* cases occur in the Democratic Republic of Congo (DRC) [23]. These data, especially for *T.b. gambiense*, illustrate an encouraging trend for countries where concerted efforts have been mounted (mainly Angola, DRC, and Sudan) to control HAT, and the decrease in incidence in recent years is due in large part to enormous efforts involving active case detection; these efforts need to be maintained despite the increasing cost per patient of detecting additional cases in control programmes that are successful [25]. There is much still to do before elimination of HAT can be considered a real option in the medium-term future. It has been estimated that up to 70,000 [24] cases actually occur annually (including *un-reported* cases); a previous estimate was 300,000 [22]; the disparities in these estimates illustrate the need for formal methods to quantify the substantial hidden burden of HAT (see below).

HAT, without distinction between *T.b. gambiense* and *T.b. rhodesiense*, was considered in the first Global Burden of Disease assessments [26], and estimated to result in 1.78 million DALYs lost across Africa, using a standard West 26 life table (life tables describe age-specific mortality in a population and determine, for example, the number of years of life lost following death at a given age), a disability weight per episode of 0.35 [27], and an annual incidence of 32,000 reported cases, including 24,000 deaths [28]. Subsequent iterations of the DALY provided revised estimates—e.g.,1.34 million DALYs lost to HAT in 2000 [29] and 1.54 million DALYs lost in 2002 [30], using an average disability-weight of 0.191 [31] and 48,511 deaths. The relative ranking of HAT to other conditions from the 2000 DALY estimates are shown in Table 1. The justification for many of the parameters pertaining to HAT and used in the Global Burden assessments (e.g., disability weighting, estimates of incidence) are not transparent and have not been published.

**Table 1:** Selected DALYs Lost by Cause, 2000.

Cause	DALYs Lost
HIV/AIDS	64,970,667
Malaria	39,568,398
Lymphatic filariasis	4,576,994
Trachoma	2,559,951
Leishmaniasis	1,752,384
Schistosomiasis	1,485,408
Ascariasis	1,405,795
**HAT (trypanosomiasis)**	**1,335,075**
Trichuriasis	803,111
Japanese encephalitis	604,002
Chagas disease	574,644
Dengue	542,954
Onchocerciasis	427,440
Leprosy	188,542
Hookworm disease	64,048

Data from [97].

## Issues in Assessing the Burden of HAT

Quantifying the impact of a disease—its burden—is a necessity in providing an evidence base for effective decision making in relation to planning of control and interventions [32]. Burden can be measured in terms of impact at a range of scales—the individual, family groups, society at large. For decision-making at the societal level (e.g., government policy, national or regional budgetary allocation, etc.), a societal, or population-based approach, is most appropriate. For this, a range of tools are available [33],[34]; the DALY is a useful and now well-established measure [35]–[37]. Proper quantification matters greatly to the neglected diseases, because a primary reason for their neglect is that their true impact on society is not known. For focal diseases such as HAT, it is necessary to choose an appropriate scale at which the assessment of burden is carried out—in many sub-Saharan African countries, the national level burden of malaria, for example, will exceed, by orders of magnitude, that of HAT, leishmaniasis [38], cysticercosis [39], or many other neglected infections. However, within a province or district, where transmission of a neglected disease occurs, it may assume a much greater importance; as budgetary decisions are increasingly made at such decentralised levels [40],[41], it is also appropriate to measure the burden of disease at this level [42],[43]. Scientific research at a range of geographic scales that uses recognised health metrics as an outcome can therefore assist in the development of effective policy.

While it is important to determine disease burden at a range of temporal and spatial scales, there are some pitfalls in doing so; at small (e.g., local) scales, annual stochastic variations in burden, resulting from annual variations in incidence, may be large and care should be taken not to over-interpret them. Also, HAT not only occurs focally, but occurs in both endemic and epidemic situations. While relative disease burdens during periods of endemic and epidemic periods matter in themselves, it would be a mistake to calculate a DALY for an epidemic and assume that this was then the more general baseline level of burden for longer-term planning. Rather, when determined for epidemics or outbreaks (e.g., [44]), this should be explicit. Routine data collection during periods of endemic transmission may be lacking in many settings, while epidemics or outbreaks focus attention on a disease, resulting in greater availability of data [45],[46]. Epidemic situations do, however, present a particular set of conditions for the assessment of the cost-effectiveness of control/long-term investment [47],[48], and DALYs, as an outcome measure of such analyses, can add significantly to the valuation of alternate investment options. The time units chosen for burden assessments also matter, particularly if outputs influence resource allocation. Recent decreases in the number of reported HAT cases [23] could, for example, lead to a reduction in effort in detecting future cases, a situation which has previously led to disease resurgence [49],[50]. It becomes important that temporal trends in incidence are accounted for in the choice of time period for assessing burden, though the sporadic nature of data availability may make this difficult in practice.

Other issues arise with regional and local studies; when conducting evaluations of disease burden within a region (e.g., relative burden of malaria and HAT in East Africa), it is appropriate to use life tables that relate to the population under study [27],[51],[52]. Indeed, country-specific life tables are produced by WHO (see http://www.who.int/whosis/database/life_tables/life_tables.cfm). However, doing so restricts the comparability of estimates between sites [53]; thus, global DALY estimates are produced using the West 26 Model Life Table [27]. For HAT, which exists only in sub-Saharan Africa, this is less of a problem, and for regional studies, using regional life tables may be more appropriate as doing so does not over-estimate burden in these communities [54]. To address these issues, a concerted research effort is required to carry out finer scale studies in a range of HAT foci that be justifiably generalised to HAT transmission zones as a whole.

### Economic Burden of HAT

We do not aim to provide a thorough review of economic studies of HAT; however, while DALYs, and other measures, in themselves allow adverse health outcomes to be rated against each other, a more practical (and intended) use of such measures is as an outcome in cost-effectiveness analyses [51],[55] (in terms of, for example, dollars spent per DALYs averted). We must ask, therefore, how cost-effective it is to control and treat HAT. Useful in this context is the rule of thumb that a cost of US$150 per DALY averted and US$25 per DALY averted is “attractive” and “highly attractive”, respectively [56]. Unsurprisingly, studies are few and far between for HAT, and have tended to focus on *T.b. gambiense*. DALYs have been used as an outcome measure in analysing the cost-effectiveness of treatment options for *gambiense* HAT [57]. Shaw and Cattand [25] illustrate that above a prevalence of approximately 2%, it becomes highly attractive to screen for and treat *gambiense* HAT using mobile teams carrying out active surveillance (the use of mobile teams is reviewed elsewhere [58],[59]). At lower prevalences, active screening may not be cost-effective in the short term, emphasising that control efforts for this disease must take a long-term perspective. Others conclude that mobile teams have too poor a coverage compared to well-trained community health workers [60]. Lutumba et al. [61] quantified the cost-effectiveness of control activities in terms of DALYs averted in Buma (Democratic Republic of Congo), a *T.b. gambiense* focus. In a population of 1,300, an active case finding activity resulted in 1,408 DALYs averted, for a cost of US$17 per DALY averted.

For *T.b. rhodesiense*, hospital-based interventions alone have been shown to be cost-effective for HAT control in rural settings in Uganda, with a mean cost per DALY averted (for reported cases) of US$8.50 [44]. Compared to hospital-based treatment of many other infections (e.g., cutaneous leishmaniasis in Colombia [48], where the cost per averted DALY was in the region of US$15,000), this is highly cost-effective. Such calculations do not generally include indirect costs to the household, which have been considered in a few studies and found to be substantial [17],[18],[61]. Devising effective methods to maximise hospital attendance and reduce the number of unreported cases in the community should thus be a priority.

The economic burden of livestock trypanosomiasis has also been reviewed [62]–[64]; for a range of neglected zoonoses, applying treatments to the animal reservoir specifically as a public health measure has been shown to be cost-effective [65], with great added benefits when these integrated interventions also improve animal health and productivity. Currently, a large-scale cattle-targeted intervention is being implemented in Uganda [66] to control the spread of *T.b. rhodesiense*. Quantifying the dual burden of infections to livestock and humans, and the added benefits to both when control is implemented, is an under-researched area and requires novel metrics and systematic data gathering across the range of neglected zoonotic diseases [65].

### Disability

DALYs consist of two major, additive components: a metric for summing mortality in a population, Years of Life Lost due to death with a condition (YLL), and a metric for summing morbidity in a population, Years of Life Lived with a Disability from a condition (YLD). The important component of YLDs is the disability weighting associated with a condition; the sum of time spent in a condition for each age group in a population is multiplied by this weighting to determine the scale of morbidity due to the condition in that population. Ideally, this is done for each of the components of morbidity—the sequelae—associated with the disease. Thus, for Chagas disease (due to infection with *Trypanosoma cruzi*), YLDs are made up of episodes plus specific sequelae relating to the incidence of cardiac complications resulting from infection, each of which has a disability weighting and an estimate for duration.

Up to the present, a weighting of 0.35 [27] and later 0.191 [67] have been used for HAT, irrespective of whether *T.b. gambiense* or *T.b. rhodesiense* HAT are being considered. No sequelae are formally listed in the disease definition for HAT used for DALY inputs [28], with the unit of measure being an episode (case definition: “Infection with protozoa of the genus *Trypanosoma*, excluding *T. cruzi*” [68]), with a standard duration of 5 years per episode (this has been recently revised for the 2004 Global Burden of Disease revision such that the length of a *T.b. gambiense* episode is 5 years and *T.b. rhodesiense* 1 year duration (C. Mathers, personal communication), recognizing that the clinical syndromes associated with infection with the different parasites have very different durations [69]). While broad differences between *T.b. gambiense* and *T.b. rhodesiense* have thus been accounted for, DALY estimates for HAT have not attempted to account for sequelae [70]–[76]. A range of sequelae should be considered for infection with both parasites—see Box 1 and Text S1—although data are lacking to enable estimates of the incidence of these sequelae to be extrapolated to the affected population at large. The stage of disease should equally be distinguished [44]: in the absence of treatment, both *T.b. gambiense* and *T.b. rhodesiense* have an early and late stage. The early stage is a febrile illness, while the second stage is defined by cerebrospinal fluid parameters—an elevated leukocyte count (>5 cells/mm^3^) or high protein levels (>37 mg/100 ml) or parasites in the cerebrospinal fluid [22],[77]. Late stage disease is neurological [78], culminating in coma and death in the absence of treatment.

**Box 1. Summary of Sequelae Associated with HAT; see Text S1 for More Details**

**Table:** 

Early stage	Non-specific signs that can include skin lesions, chancre, pruritus, and cardiac, endocrine, and gastrointestinal problems.
Late stage (parasite infection of the central nervous system)	Tremors, motor weakness, walking difficulties, sensory disorders, visual impairments, headache, sleep disturbances that deteriorate into coma.
Early stage treatment	Suramin: Reactions depend on overall patient condition, with severe reactions in <5% of patients, and include pyrexia and mild nephrotoxicity, kidney damage, collapse with nausea, vomiting, shock, delayed hypersensitivity reactions (e.g., exfoliative dermatitis), severe diarrhoea, and jaundice.
	Pentamidine: Hypotensive reactions, and damage to liver, kidneys, and the pancreas.
Late stage treatment	Encephalopathy occurs in 5%–10% of treated cases, with a mortality rate of 10%–70%. Convulsions, progressive coma, or psychotic reactions. Acute haemorrhagic leuco-encephalopathy associated with progressive coma and hypoxic brain damage with convulsions or heart failure. Other effects of melarsoprol include liver toxicity, severe enterocolitis, fatigue, arthralgia, myalgia and fever, pruritus, urticaria, and gastrointestinal reactions; cardiovascular side effects such as tachycardia, palpitations, chest pain, hypotension, and phlebitis were reported.
	A relapse rate of 3%–10% is commonly reported; however, high failure rates have been reported recently from *T.b. gambiense* areas of some countries.
	Eflornithine treatment frequently results in side effects, although they are less severe than melarsoprol-induced encephalopathy, and usually reversible. Reactions include convulsions, gastrointestinal symptoms, bone marrow toxicity resulting in anaemia, leucopenia and thrombocytopenia and alopecia, fatigue, arthalgia, dizziness, insomnia, fever, headache, and anorexia.
Long-term sequelae beyond cure	Few data, with confounding between impacts of infection and treatment. Possible growth retardation and neurological impairment.

**Figure 2:**
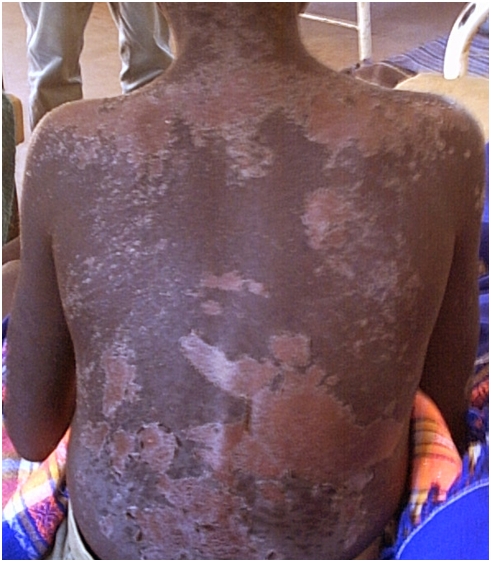
Child with Resolved Exfoliative Dermatitis (Epidermal Necrolysis) Resulting from Early Stage *T.b. rhodesiense* HAT Treatment with Suramin. The acute stage of this condition presents with blistering and the peeling off of large areas of skin; complications due to secondary infection are likely. Image credit: Eric Fèvre.

Morbidity induced by treating HAT has also not been taken in to account in burden calculations: the drugs to treat HAT are generally toxic [79],[80], with a relatively high proportion of side effects (e.g., exfoliative dermatitis in 1% of treatments with suramin [see Figure 2]; melarsoprol-induced encephalopathy in 5%–10% of patients). For other diseases, morbidity resulting from treatment has been included in burden estimates, e.g., vaccine reactions account for approximately 2.5% of the overall DALY score for rabies in Asia and Africa [81]. There is also some evidence of long-term impacts of *T.b. gambiense* HAT infection beyond parasitological cure [82],[83], but the significance of this at a population level remains to be quantified by the HAT research community, and there are no similar studies for *T.b. rhodesiense*.

### Under-Reporting

A significant limitation of incidence figures, and subsequent estimates of burden based on these, published through the World Health Organization [24] and originating from national bodies (e.g., ministries of health), is that they relate only to reported deaths. HAT cases are itemised when they are identified in community screening exercises (active detection) or passively in hospitals. In common with most causes of death in Africa [84], non-hospitalised HAT cases are not recorded, and patients are often unable to afford to present for treatment [18]. For *T.b. rhodesiense*, studies based on quantifying under-reporting using data on the early∶late stage ratio [85] have shown that between 38% and 41% of *T.b. rhodesiense* cases go un-reported [85],[86] in Uganda, with a similar picture in Tanzania (L. Matemba,personal communication). For *T.b. gambiense*, there have been preliminary attempts to estimate this directly [61]; Robays et al. [59] used a Piot modelling approach and highlighted that many cases may be missed during active screening programmes for *T.b. gambiense* (partly due to test sensitivity); cases will, of course, also be missed where there are no case-detection programmes on-going, and clearly, cases will be missed where active detection activities are not on-going. . Un-reported cases go untreated and almost invariably result in death. Existing estimates of under-reporting need to be validated in a range of settings before they can be confidently extrapolated, however, as there may be site-specific influences on the magnitude of this parameter. With few exceptions (e.g., leishmaniasis [87], *T.b. rhodesiense* HAT [85], rabies [81],[88]), under-reporting rates have rarely been properly quantified for neglected diseases, so progress in this regard for HAT is promising. Unfortunately, health care systems themselves may even be missing many cases of HAT on presentation at non-specialist units [17], while in some settings communities may be aware of the disease and its dangers but not report cases, as they are aware that drugs and treatment are not available in health units [89]. A greater research effort is required to investigate the impact of these factors on reporting rates in different parts of Africa, and this research needs to translate to activities to remedy the situation at country and local levels. Across the continent, we have seen that a figure of 70,000 cases per year is cited; if this is even roughly accurate, it recognises that approximately 50,000 HAT cases may be undetected, and thus die (unaccounted for in the burden calculations), in any given year.

## Challenges and Future Steps

Akin to the challenges involved in the assessment of burden of most neglected tropical diseases, data on HAT incidence, morbidity, and mortality is incomplete and fragmented at present. Under-reporting of HAT, exacerbated by insufficient access to health care by patients, as well as confounding with concurrent endemic diseases such as malaria and HIV/AIDS, is a significant obstacle. Methods to quantify levels of under-reporting of both *T.b. gambiense* and *T.b. rhodesiense* [59],[85] need to be validated and extended to foci in different countries. As well as estimating mortality, those living in HAT foci must be enumerated to provide a denominator for incidence figures; estimates of the population at risk, validated by field data, are urgently required. This would enable the limited resources available for data collection and public health interventions to be deployed as efficiently as possible. We have seen that morbidity associated with HAT is currently represented by single, average disability weightings in global comparative assessments. This does not reflect the dual causation of HAT (*gambiense* and *rhodesiense*), the distinction between early and late stages (and the reduction in the societal burden that can be achieved by early detection of cases), or treatment-associated morbidity. While alternative disability weightings for use in DALY calculations have been proposed and used [44], a wider consultative exercise is necessary to reach a data-driven consensus.

This review has largely concentrated on the DALY as a metric for assessing the burden of HAT. There have been many criticisms of this measure [90],[91], and its value in assessing the burden of neglected diseases specifically has recently been questioned [20],[92]. Other measures that correct issues in the DALY metric should be developed, but for the short to medium term, the DALY is firmly in place as the metric of choice, with a range of refinements, particularly in terms of transparency of inputs, planned for the next iteration of the Global Burden of Disease project [31]. Importantly for HAT and other neglected diseases, simply changing the metric will not address the core issues: tackling neglect in relation to understanding disease epidemiology, at a range of spatial scales, including collecting data to make better decisions about control and provide material for advocacy. Who is at risk? Where are the cases? How many are there? How much do they suffer? How many people die with un-diagnosed infection? What co-factors impact on the burden of infection and what co-morbidities [93] does HAT share with other, concurrent infections? Will it be cost-effective to deploy novel therapies [94]? These are not new questions, but contemporary answers are lacking. Importantly, answering them is a fundamental first step in the proper assessment of the burden of disease and in providing an evidence base for measuring the success of existing and future HAT control initiatives. Existing databases should be mined to extract data that helps answer these questions, and funding must be made available to address these issues appropriately where data do not already exist. The HAT research community appears to have mobilised to address the neglect in the development of therapeutics for the disease [95],[96], and must take up the equally important challenge of better understanding the impact of this infection in affected populations.

**Box 2. Key Learning Points**

1. HAT is a neglected tropical disease with two causative organisms, *Trypanosoma brucei rhodesiense* and *T.b. gambiense*; the burden of HAT must account separately for infection with these two parasites.

2. The true burden of HAT is poorly reflected in many existing assessments, as is the case with other neglected diseases.

3. HAT burden assessments need to account for parasite-specific, disease stage–specific, and treatment-related morbidity.

4. The rate of HAT under-reporting is as high as 40% in some *T.b. rhodesiense* foci; under-reporting has not been formally quantified for *T.b. gambiense*.

5. The population at risk from HAT needs to be quantified to serve as a denominator for incidence calculations.

## Supporting Information

Text S1 Online Appendix: Sequelae
(0.04 MB PDF)
