# Supplementary material for: The Burden of Human African Trypanosomiasis
Source: PLoS Negl Trop Dis. 2008 Dec 23;2(12):e333. doi: 10.1371/journal.pntd.0000333 (PMC2602597; doi:10.1371/journal.pntd.0000333)
Supplement: Text S1 — Online Appendix: Sequelae (0.04 MB PDF) [file pntd.0000333.s001.pdf]

Supplementary Information (S1): The burden of Human African Trypanosomiasis: Sequelae

E.M. Fèvre, B.v. Wissmann, S.C. Welburn, P. Lutumba

Introduction

Our manuscript has addressed a range of issues in the calculation of the burden of human African trypanosomiasis (HAT) caused by both *T.b. gambiense* and *T.b. rhodesiense*. Our aim in this appendix is to provide a fuller picture of the range of sequelae that may be suffered by a patient infected with either of the causative organisms of HAT, and to illustrate some of the treatment-associated sequelae that need to be more fully considered in the calculation of the human disease burden. This appendix does not aim to provide a review of diagnostic clinical signs that might be seen and drug therapies that may be used in a HAT case; for that purpose, we direct the reader to a range of other studies [1-9].

1st stage – (haemolymphatic stage)

In the first stage of sleeping sickness, the initial systemic clinical signs are often non-specific and include fever, general malaise, facial oedema, anaemia, lymphadenopathy and arthralgia. As the disease progresses, clinical signs will reflect the involvement of various organ systems. Pathology can include a range of symptoms some of which may be rare: skin lesions in addition to the chancre, pruritus, cardiac problems (heart failure, myocarditis, endocarditis, ECG changes), endocrine problems (resulting in impotence, gynaecomastia, hair loss, menstrual irregularities and reproductive dysfunction), gastrointestinal problems and in some cases eye-related pathology [10].

Lymph and circulatory system

Lymph is the preferred site for trypanosomes manifesting in swollen lymph nodes. Swelling of the cervical lymph nodes, commonly referred to as “Winterbottom’s sign” is an important diagnostic feature of Gambian sleeping sickness [8]. The high metabolic demands of periodically arising, rapidly dividing new antigenic variants of trypanosomes are met in the blood stream. At intervals of several days to a month, waves of parasitaemia occur throughout the course of infection, which may last from a day to a week and are associated with high fever. The blood composition is disturbed, and erythrocytes and white blood cell counts are reduced. During rapid expansion of a variant surface glycolprotein (VSG)-variant, B-cells are stimulated through both T-cell dependent and independent pathways. An increase in immunoglobulins, especially IgM, eliminates the majority of trypanosomes, with only heterologous antigen variants surviving to repopulate the blood and other tissues.

Paradoxically, despite generally increased responsiveness of B and T cells, sleeping sickness is associated with progressive immunosuppression due to impairment of T-cell responses [9].

The heart is considered at high risk, especially in *T. brucei rhodesiense* infections. In many patients there is evidence of damage from a range of abnormalities including tachycardia, bradycardia, cardiac murmurs, myocardiatis with congestive heart failure, pericarditis, pericardial effusions and pancarditis [8]. A recent case-control study on second stage *T. b. gambiense* patients, suggested significantly higher prevalence of major ECG alterations in sleeping sickness patients (n=60) than controls, but cardiopathy rarely resulted in severe congestive heart failure and subsided after treatment [11]. Increased capillary fragility and permeability, resulting in petechial haemorrhaging is also associated with generalized oedema, especially in the extremities and the face, where sub-orbital puffiness was once considered a key diagnostic sign of sleeping sickness.

Pathological changes in other organ systems

Tissue barrier damage in kidneys glomeruli also results in pathological loss of a number of substances from serum [9]. Spleen enlargement correlating to the anaemia is frequently observed, with sinusoids becoming packed with active macrophages [8].

Skin rashes affecting the shoulders, trunk and thighs are common [9] and intense pruritus is present in about one third of sleeping sickness patients in the first stage of the disease [12,13]. There is generalized skeletal muscle atrophy with patchy cellular infiltration, often manifesting in the diaphragm [9]. Gastrointestinal damage may be evident. Laboratory models of *T. b. brucei* infections demonstrated marked structural changes in the intestine: reduction in villous height, wall thickness and oedema in particular in the jejunum – associated with increased intestinal leakage.

Hormone and endocrine changes occur in a high proportion of patients, and may manifest in menstrual disorders, sterility, susceptibility to abortions or premature births, stillbirths and perinatal death. This hormonal imbalance can also be associated with uterine hypoplasia and atrophy of sexual organs. In men, changes result in impotence and testicular atrophy, and in the later stages gynaecomastia (fat tissue adopts female distribution). These changes are manifestations of profound underlying changes in hormonal function. At the gross level, adrenals and thyroid are infiltrated and atrophy with time. Some hormonal changes in the later stage of sleeping sickness are initiated by dysfunction of the circadian pacemaker system which is intricately linked to a range of hormone systems [9].

In some cases, pathology of the eyes such as iritis, keratitis, conjunctivitis and choroidal atrophy is reported, but it is unclear whether these are directly attributable to the trypanosome

infection, or occur as a result of general immunosuppression facilitating concurrent bacterial infection [9].

2nd stage (meningo-encephalic stage)

The second stage of sleeping sickness is defined by diagnostic evidence of trypanosome invasion of the central nervous system (CNS). Most functions of the CNS can be affected by pathology, and the patient can experience a wide range of mental, psychiatric as well as neurological dysfunctions. CNS involvement can start within a few weeks or several months of initial infection (a study of duration of symptoms of Rhodesian sleeping sickness in south-east Uganda reported involvement of the CNS within between 3 weeks and 2 months of infection [7]) or sometimes even years in the case of *T.b. gambiense* infections [14]. However onset of insidious neurological signs and mental disturbances may occur before detectable changes in the cerebro-spinal fluid [9]. Neurological symptoms are typical for the second stage of sleeping sickness, whereas fever is only rarely observed (<1/5 patient) [6].

Clinical signs experienced include the characteristic sleep disturbances, which originally gave the disease its name, and which are the leading symptom in the published literature, suffered by approximately three-quarters of late stage patients [6]. Late stage sleeping sickness patients have frequent, short sleep episodes throughout the day and night, with the total time of sleep being equal to that of a healthy person as shown by somnographic studies reporting irregularities of the sleep-wake cycle rather than hypersomnia [15,16]. In the final stages, sleep-episodes deteriorate into coma, which finally results in death, if the patient remains untreated.

Other symptoms associated with the meningo-encephalic stage of sleeping sickness, as described by a large scale study of over 2500 patients (16 treatment centres in 7 sub-Saharan countries (Angola, Central African Republic, Cote d'Ivoire, Democratic Republic of Congo, Equatorial Guinea, Republic of Congo and Southern Sudan)) include tremors (21%), motor weakness (34.8%), abnormal movements (10.5%), walking difficulties (21.7%) speech disorder (13.4%) and unusual behaviour (24.7%) [6]. Psychiatric symptoms increased significantly (5 to 42%), with increasing duration of disease and CSF white blood cell counts [6]. Sensory disorders like hyperaesthaesia (17% [13]) or paraesthesia (15% [12]) have also been reported for second stage patients. Visual impairments have also been reported [10]. Headache is a frequent (78.8%) but unspecific symptom, which occurred at similar frequencies throughout both stages of the disease [6]. Large variations in the proportion of patients affected by various signs are typical in the published literature, and may be attributable to small study populations, concurrent infections or geographical variation [6].

Adverse effects of suramin treatment of first stage sleeping sickness

Adverse drug reactions to suramin are dependent on the general clinical condition of the patient, including nutritional status and co-infection (especially with onchocerciasis). Adverse reactions other than pyrexia and mild nephrotoxicity, which is usually reversible, are rare. Malnourished patients are particularly at risk of kidney damage. Other adverse reactions are reported at prevalences of <5% (although underreporting can not be excluded). Immediate life threatening adverse effects of suramin include collapse with nausea, vomiting, shock and urticaria (typical signs of early hypersensitivity). Delayed hypersensitivity reactions include exfoliative dermatitis (epidermal necrolysis [17]), bone marrow toxicity with agranulocytosis, haemolytic anaemia, thrombocytopenia and reactive encephalopathy, a number of which can also be life-threatening [18,19]. Severe diarrhoea and jaundice have also been described [18]. Adrenocortical degeneration and insufficiency have been reported in connection with high but not normal dose treatment.

Adverse effects of pentamidine treatment of first stage sleeping sickness

Administration of intravenous pentamidine can result in severe hypotensive reactions (therefore intramuscular injection is used preferentially). Damage can also ensue to liver, kidneys and the pancreas, with diabetes a possible long term effect of pancreas damage [18].

Adverse effects of melarsoprol treatment of second stage sleeping sickness

Encephalopathy is the most life threatening complication of melarsoprol treatment of second stage human African trypanosomiasis. Encephalopathy occurs in 5-10% of all melarsoprol treated cases, with a mortality rate of 10-70% [20,21]. It has been reported that encephalopathy may be rarer but more severe in younger age groups [22]. The clinical signs have been defined as either convulsions, a progressive coma or psychotic reactions and abnormal behaviour [20]. No association between encephalopathy and severity of trypanosome induced meningo-encephalitis has been found. Acute haemorrhagic leuco-encephalopathy was associated with progressive coma and hypoxic brain damage with convulsions or heart failure [2,23].

Other adverse effects of melarsoprol including liver toxicity, severe enterocolitis and diffuse peripheral neuropathy can also be life-threatening [24]. General side effects can include fatigue, arthralgia, myalgia and fever. Cutaneous reactions including pruritus, urticaria, amongst others have been observed. Cardiovascular side-effects such as tachycardia, palpitations, chest pain, hypotension and most commonly phlebitis were reported. Gastrointestinal reactions observed included anorexia, nausea, vomiting abdominal pain, diarrhoea and epigastric pain. Predominant neurological signs included headaches, dizziness and tremors [25].

A relapse rate of 3-10% is commonly reported with melarsoprol treatment, however recently high failure rates have been reported from *T.b. gambiense* areas of northern Uganda 30% [26], Sudan: 16-21% [27], Angola and Congo 25% [28].

Adverse effects of eflornithine treatment of second stage sleeping sickness

Eflornithine treatment frequently results in side effects, similar to those of other cytotoxic drugs, although they are less severe than melarsoprol induced encephalopathy. Eflornithine side effects are generally reversible on conclusion of treatment [29,30]. Duration of treatment and the general condition of the patient influence the occurrence and severity of side-effects. These include convulsions (7%) [31], gastrointestinal symptoms (vomiting, nausea, diarrhoea) (10-39%), bone marrow toxicity resulting in anaemia, leucopenia and thrombocytopenia (25-50%) [32] and alopecia (5-10%) . Other side-effects reported include fatigue, arthalgia, dizziness, insomnia, fever, headache and anorexia [33].

Cure rates of 91% have been reported [34]. However as eflornithine is a trypanostatic drug and needs a competent immune system for clearance of infection, eflornithine can not cure HIV-positive sleeping sickness patients [35].

Long term sequelae as a result of infection and treatment

There are very few studies on the longer term impacts of HAT, and published evidence concentrates on *T.b. gambiense*. Aroke *et al.* [36] found that children treated (with melarsoprol) for late stage HAT scored lower on growth measures and had lower academic performance than matched, non-affected controls. Others have shown that melarsoprol treatment of children results in neurological disorders beyond parasitological cure [37].

References

1. Moore DAJ, Edwards M, Escombe R, Agranoff D, Bailey JW, et al. (2002) African trypanosomiasis in travelers returning to the United Kingdom. Emerging Infectious Diseases 8: 74-76.
2. Adams JH, Haller L, Boa FY, Doua F, Dago A, et al. (1986) Human African trypanosomiasis (*T.b. gambiense*): a study of 16 fatal cases of sleeping sickness with some observations on acute reactive arsenical encephalopathy. Neuropathology and Applied Neurobiology 12: 81-94.
3. Legros D, Ollivier G, Gastellu-Etchegorry M, Paquet C, Burri C, et al. (2002) Treatment of human African trypanosomiasis — present situation and needs for research and development. Lancet Infectious Diseases 2: 437-440.

4. Dumas M, Bisser S (1999) Clinical aspects of human African trypanosomiasis. In: Dumas M, Bouteille B, Buguet A, editors. Progress in Human African Trypanosomiasis, Sleeping Sickness. Paris: Springer. pp. 215-233.
5. Bouteille B, Oukem O, Bisser S, Dumas M (2003) Treatment perspectives for human African trypanosomiasis. Fundamental & Clinical Pharmacology 17: 171-181.
6. Blum J, Schmid C, Burri C (2006) Clinical aspects of 2541 patients with second stage human African trypanosomiasis. Acta Tropica 97: 55-64.
7. Odiit M, Kansiime F, Enyaru JCK (1997) Duration of symptoms and case fatality of sleeping sickness caused by *Trypanosoma brucei rhodesiense* in Tororo, Uganda. East African Medical Journal 74: 792-795.
8. Apted FIC (1970) Clinical manifestations and diagnosis of sleeping sickness. In: Mulligan HW, Potts WH, Kershaw WE, editors. The African Trypanosomiases. London: George Allen and Unwin. pp. 661-683.
9. Pentreath VW, Kennedy PGE (2004) Pathogenesis of Human African Trypanosomiasis. In: Maudlin I, Holmes PH, Miles MA, editors. The trypanosomiases. Wallingford, Oxfordshire: CABI Publishing.
10. Kennedy PGE (2006) Diagnostic and neuropathogenesis issues in human African trypanosomiasis. International Journal for Parasitology 36: 505-512.
11. Blum JA, Burri C, Hatz C, Kazumba L, Mangoni P, et al. (2007) Sleeping hearts: the role of the heart in sleeping sickness (human African trypanosomiasis). Tropical Medicine and International Health 12: 1422-1432.
12. Ginoux C, Frezil JL, Alary JC (1982) Symptoms of human trypanosomiasis at the first diagnostic phase in the People Republic of Congo (author's translation). Med Trop (Mars) 42: 281-287.
13. Boa YF, Traore MA, Doua F, Kouassi-Traore MT, Kouassi BE, et al. (1988) Les différents tableaux cliniques actuels de la trypanosomiase humaine africaine a T. b. gambiense. Analyse de 300 dossiers du foyer de Daloa, Cote d'Ivoire. Bulletin de la Société de Pathologie Exotique 81: 427-444.
14. Checchi F, Filipe JA, Haydon DT, Chandramohan D, Chappuis F (2008) Estimates of the duration of the early and late stage of gambiense sleeping sickness. BMC Infectious Diseases 8: 16.
15. Buguet A, Tapie P, Bert J (1999) Reversal of the sleep/wake cycle disorder of sleeping sickness after trypanosomicide treatment. Journal of Sleep Research 8: 225-235.
16. Buguet A, Bert J, Tapie P, Tabaraud F, Doua F, et al. (1993) Sleep-wake cycle in human African trypanosomiasis. Journal of Clinical Neurophysiology 10: 190-196.
17. May E, Allolio B (1991) Fatal toxic epidermal necrolysis during suramin therapy. European Journal of Cancer 27: 1338.
18. Fairlamb AH (2003) Chemotherapy of human African trypanosomiasis: current and future prospects. Trends in Parasitology 19: 488-494.
19. Burri C, Stich A, Brun R (2004) Current chemotherapy of Human African trypanosomiasis. In: Maudlin I, Holmes PH, Miles MA, editors. The trypanosomiases. Wallingford, Oxfordshire: CABI Publishing.
20. Pépin J, Milord F (1994) The treatment of human African trypanosomiasis. Advances in Parasitology 33: 1-47.
21. World Health Organization (1998) Control and Surveillance of African Trypanosomiasis. Geneva: WHO. 114 p.
22. Eperon G, Schmid C, Loutan L, Chappuis F (2007) Clinical presentation and treatment outcome of sleeping sickness in Sudanese pre-school children. Acta Tropica 101: 31-39.
23. Haller L, Adams H, Merouze F, Dago A (1986) Clinical and pathological aspects of human African trypanosomiasis (*T. b. gambiense*) with particular reference to reactive arsenical encephalopathy. American Journal of Tropical Medicine and Hygiene 35: 94-99.
24. Chappuis F (2007) Melarsoprol - free drug combinations for second-stage Gambian sleeping sickness. Clinical Infectious Diseases 45: 1443-1445.
25. Bisser S, N'Siesi X, Lejon V, Preux PM, Van Nieuwenhove S, et al. (2007) Equivalent trial of melarsoprol and nifurtimox monotherapy and combination therapy for the treatment of second stage *Trypanosoma brucei gambiense* sleeping sickness. Journal of Infectious Diseases 195: 322-329.

26. Legros D, Evans S, Maiso F, Enyaru JCK, Mbulamberi D (1999) Risk factors for treatment failure after melarsoprol for *Trypanosoma brucei gambiense* trypanosomiasis in Uganda. Transactions of the Royal Society of Tropical Medicine and Hygiene 93: 439-442.
27. Brun R, Schumacher R, Schmid C, Kunz C, Burri C (2001) The phenomenon of treatment failures in Human African Trypanosomiasis. Tropical Medicine and International Health 6: 906-914.
28. Stanghellini A, Josenando T (2001) The situation of sleeping sickness in Angola: a calamity. Tropical Medicine and International Health 6: 330-334.
29. Burri C, Brun R (2003) Eflornithine for the treatment of human African trypanosomiasis. Parasitology Research 90: S49-S52.
30. Chappuis F, Udayraj N, Stietenroth K, Meussen A, Bovier PA (2005) Eflornithine is safer than Melarsoprol for the treatment of second-stage *Trypanosoma brucei gambiense* Human African Trypanosomiasis. Clinical Infectious Diseases 41: 748-751.
31. Blum J, Nkunku S, Burri C (2001) Clinical description of encephalopathic syndromes and risk factors for their occurrence and outcome during melarsoprol treatment of human African trypanosomiasis. Tropical Medicine and International Health 6: 390-400.
32. Anonymous (1991) Ornidyl patent information. Kansas City, USA.
33. Creaven PJ, Pendyala L, Petrelli NJ (1993) Evaluation of alpha-difluoromethylornithine as a potential chemopreventive agent: tolerance of daily oral administration in humans. Cancer Epidemiology, Biomarkers and Prevention 2: 243-247.
34. Milord F, Pepin J, Loko L, Ethier L, Mpia B (1992) Efficacy and toxicity of eflornithine for treatment of *Trypanosoma brucei gambiense* sleeping sickness. Lancet 340: 652-655.
35. Pepin J, Ethier L, Kazadi C, Milord F, Ryder R (1992) The impact of human immunodeficiency virus infection on the epidemiology and treatment of *Trypanosoma brucei gambiense* sleeping sickness in Nioki, Zaire. American Journal of Tropical Medicine and Hygiene 47: 133-140.
36. Aroke AH, Asonganyi T, Mbonda E (1998) Influence of a past history of Gambian sleeping sickness on physical growth, sexual maturity and academic performance of children in Fontem, Cameroon. Annals of Tropical Medicine and Parasitology 92: 829-835.
37. Cramet R (1982) La maladie du sommeil chez l'enfant et ses sequelles a distance. A propos de 110 observations personnelles a l'hopital de Fontem (Cameroun). Médecine Tropicale (Mars) 42: 27-31.
